# Is the Pain Just Physical? The Role of Psychological Distress, Quality of Life, and Autistic Traits in Ehlers–Danlos Syndrome, an Internet-Based Survey in Italy

**DOI:** 10.3390/healthcare9111472

**Published:** 2021-10-30

**Authors:** Matteo Rocchetti, Alessandra Bassotti, Jacopo Corradi, Stefano Damiani, Gianluigi Pasta, Salvatore Annunziata, Viviana Guerrieri, Mario Mosconi, Davide Gentilini, Natascia Brondino

**Affiliations:** 1 Department of Brain and Behavioral Sciences, University of Pavia, 27100 Pavia, Italy; jacopo.corradi01@universitadipavia.it (J.C.); stefano.damiani01@universitadipavia.it (S.D.); davide.gentilini@unipv.it (D.G.); natascia.brondino@unipv.it (N.B.); 2 Regional Center of Ehlers-Danlos Syndrome, IRCCS Ca’ Granda Foundation, 20122 Milan, Italy; alessandra.bassotti@policlinico.mi.it; 3 Department of Orthopedics and Traumatology, Fondazione Policlinico IRCCS San Matteo, University of Pavia, 27100 Pavia, Italy; gianluigipasta@yahoo.it (G.P.); salvatoreannunziata89@gmail.com (S.A.); viviana.guerrieri@gmail.com (V.G.); mario.mosconi@unipv.it (M.M.)

**Keywords:** Ehler–Danlos, autism, anxiety, depression, quality of life, pain

## Abstract

Background: Ehlers–Danlos syndromes (EDS) have been associated with psychological distress, comorbid psychiatric disorders, and worsening in quality of life (QoL). Among the neurodevelopmental disorders, autism spectrum disorders (ASD) have shown the highest rates of co-occurrence with EDS. The reasons for these associations are unknown and a possible role of pain in increasing the risk of psychiatric disorders in EDS has been suggested. However, a detailed picture of an Italian EDS sample is still lacking. Methods: We conducted a web-based survey in a third level center for the diagnosis of EDS in northern Italy, to investigate psychological distress, QoL, and the presence of autistic traits. Furthermore, we correlated the psychometric data with some clinical variables. Results: We observed a high rate of psychological distress with 91% of the responders at high risk of common mental disorders, low QoL, and high prevalence of autistic traits in EDS patients. Specifically, patients lacking a specific genetic test, diagnosed as suspects of EDS appeared to be at greater risk and reported worse psychological QoL. Pain was significantly associated with both psychological distress and worse QoL. Conclusions: Our findings support the need of further research and of a multi-disciplinary approach to EDS including psychological and psychiatric liaison.

## 1. Introduction

Ehlers–Danlos syndromes (EDS) encompass a group of heterogeneous heritable connective tissue disorders associated with joint hypermobility, skin hyperextensibility, and tissue fragility [1]. Recent epidemiological studies of EDS and hypermobility spectrum disorders estimated a prevalence ranging from 1.2 over 1000 [2] to 1.9 over 1000 [3]. The current international classification of the EDS refers to 13 subtypes, diagnosed according to both clinical criteria and genetic data. Unfortunately, several patients do not obtain a precise diagnosis but receive a “provisional clinical diagnosis”, with limited genetic counselling possibility and with a high level of prognostic uncertainty, potentially enhancing the psychological burden of the patient. In fact, EDS are considered rare multisystemic disorders [4] with significant functional impairment and possible life-threatening complications [5] that differ according to the diagnostic subtype. Individuals suffering from EDS frequently receive the diagnosis later in life, reporting a troublesome path of health care seeking, with frequent diagnostic errors and loss of confidence in the health-care system [6]. Longer delays in diagnosis have been associated with psychological suffering and risk of attempted suicide [2]. According to the international classification of EDS, one of the most frequent and severe manifestations of EDS, the hypermobile subtype (hEDS) remains a purely clinical diagnosis as there is yet no reliable genetic etiology for most of the patients [1]. Remarkably, several systemic features have been described in hEDS that could be even more debilitating than joint symptoms, impairing global functioning and quality of life. Among these, both physical (orthostatic tachycardia, gastrointestinal disorders, dysautonomia) and psychiatric conditions (anxiety, depression) should be carefully evaluated during clinical assessments [1]. Unfortunately, during the path to diagnosis, people suffering from hEDS felt frequently ignored or not considered in their pain, often only receiving a psychological/psychiatric explanation of their suffering [6]. Therefore, even after diagnosis, the risk of minimizing psychological aspects or avoiding the confrontation of comorbid psychiatric disorders should be carefully considered to avoid poor treatment adherence or suboptimal treatment [7,8].

Several studies investigated the presence of psychiatric disorders in EDS, especially in the hypermobile subtype. A clear association between hEDS and anxiety has emerged [3,9]. The association has been found both in cross-sectional as well as longitudinal studies [10,11], reporting a relative risk greater than 20 of developing a panic attack disorder as compared with the general population, with a significant increase of anxiolytic medications use. Moreover, an increased risk of depression and mood disorders have been found [2,12], being at least in part mediated by the presence of anxiety [13]. Of note, physical pain could be a risk factor for psychiatric disorders in EDS, especially for depression [14].

Among the psychiatric conditions, the growing literature data suggested a strong association between EDS and neurodevelopmental disorders [15]. Autism spectrum disorders (ASD) and attention-deficit/hyperactivity disorder (ADHD) had a relative risk of 7.4 and 5.6, respectively in people suffering from EDS compared with the general population in a Swedish nationwide cohort study [2].

The underlying mechanism for the association between psychiatric conditions and EDS is unknown, but several theories have been formulated ranging from shared genetic risk, autonomic disfunction, decreased proprioception, and chronic pain syndrome [7]. For example, a few authors suggested a strong association of psychiatric disorders in EDS with pain [16].

Following from these premises, quality of life (QoL) has been found as significantly reduced in EDS subjects compared with controls [17] and the general population [18]. However, there is still a paucity of data regarding mental health issues in EDS, especially in the Italian population [19,20]. We aimed at investigating the psychological distress, the QoL, and the presence of autistic traits in subjects referred through a third level center for the diagnosis and management of EDS in northern Italy. We specifically decided to investigate the presence of autistic traits as ASD has shown the higher relative risk of comorbidity among neurodevelopmental conditions in EDS [2]. Second, we aimed at analyzing the possible correlation between the psychometric tools and clinical variables, with a specific focus on pain, in order to identify potential risk factors.

## 2. Materials and Methods

This is an observational cross-sectional study, investigating with the use of internet-based self-report questionnaires the psychological wellbeing, the quality of life, and the presence of autistic traits, in patients referred through a third level center for the EDS in northern Italy. The study was reported according to the CHERRIES guidelines for online surveys (please see Supplementary Material section) [21].

### 2.1. Subjects

All of the adult patients diagnosed with EDS, referred through the EDS clinic (Regional Center of Ehlers-Danlos Syndrome, IRCCS Ca’ Granda Foundation, Milan, Italy), were invited to participate in this research with the use of emails and phone calls between May and June 2017. After the agreement is obtained, explanatory materials were provided and a written informed consent was acquired before sending the electronic link to complete the self-report.

Informed consent included study aims, study personnel, and information on the confidentiality of the data, anonymity procedures, and personal data protection. Before completing the online survey, participants were required to respond to all of the items. Respondents were able to review and change their answers before submitting the questionnaire. The IP address of the participant’s computer was used to identify potential duplicate entries from the same user. Additional entries for the same IP address were never allowed. The completion time for all of the items was approximately 20 min. No incentive was offered for participation. Questionnaires were distributed electronically over a 2-month period (1 May 2017–30 June 2017), and participants consenting to the study were solicited three times before exclusion. After the matching of clinical and e-survey information, the database was anonymized. The diagnosis of each participant was reviewed according to the recent published international criteria [1]. As recommended, patients that did not fulfill the criteria for any specific subtype, but with a highly probable diagnosis, were given a provisional clinical diagnosis and have been named as suspected EDS (sEDS) throughout the paper. Other acronyms are coherent with the consensus paper [1] identifying classical (cEDS), hypermobile (hEDS), and vascular (vEDS) subtypes.

### 2.2. Assessment

Clinical records were reviewed to record the gender, age, occupational status, and presence of a disability benefit. Furthermore, the approximate age of symptoms onset (<4 years 4–12 years, 13–17 years, 18–26 years, >27 years), age at diagnosis, frequency of articular luxation (<=1/year, 2–12/year, >12/year), presence of arthralgia, and intensity of pain were extracted.

Three questionnaires were sent to the participants.

The Global Health Questionnaire (GHQ-12) is a self-rated tool that evaluates the general mental and physical health, through 12 questions rated with the Likert method. It is considered the gold standard screening tool for affective disorders in patients’ populations and is widely used as a measure of common mental disorders in public health surveys. The GHQ-12 has been validated in Italy [22], and the authors suggested a Likert cut-off >8. However, we decided to adopt a conservative cut-off ≥14, that in recent research provided the best area under the curve in discriminating healthy controls and individuals with mental disorders [23], showing excellent discriminant validity.

The Autism Quotient (AQ) is a self-administered questionnaire, developed to reveal the presence of autistic traits in the general population. Fifty questions provide a score ranging between 0 and 50, and a cut-off ≥32 is considered suggestive of autistic traits that deserve further investigation [24].

Quality of life (QoL) was investigated through the World Health Organization Quality of Life, short version (WHOQOL-BREF), an internationally and cross-culturally validated quality of life assessment tool. There are two general questions and four QoL domains: Physical health, psychological, social relationship, and environment. Each domain score is corrected to be comparable with the extended WHOQOL scale and to range from 0 to 100. For each domain, we referred to the mean reported by Skevington et al. [25] in the field trial and decided to consider a significant reduction in the QoL of the individuals who scored <70, according to Cummins [26] and recent research [27,28]. Finally, in order to identify subjects with the worse QoL for each domain, we considered the QoL to be severely impaired when the score was one standard deviation (SD) below the mean of our sample [29].

Intensity of pain at the last visit was measured according to a visual analog scale (VAS) ranging from 0 to 10 [30].

### 2.3. Statistical Analysis

Continuous and discrete variables derived from the self-assessment tools have been reported with descriptive statistics (means, standard deviations (SD), frequencies with %). After controlling for normal distribution and homogeneity of variance, the ANOVA test was performed to compare continuous variables in different EDS subgroups, and Fisher’s exact test was used for categorical variables. Post-hoc multiple comparisons, corrected with Bonferroni’s method, were then performed when appropriate. Furthermore, Pearson’s correlation coefficient was used to understand the possible role of pain in increasing the risk of common mental disorders, worsening quality of life, and autistic traits. Each statistic was considered significant with two-tailed *p* < 0.05. SPSS 26.0 (IBM Corp., Armonk, NY, USA) was used for all of the calculations.

The study has been approved by our internal review board.

## 3. Results

At the end of the recruitment, 515 patients referred through the ESD clinic were contacted. Twelve refused to participate, 347 did not answer within the predefined temporal framework, and 156 patients provided a written informed consent and completed the survey. The included sample did not differ by age and gender from the patients that did not answer the survey. Most of our sample were female (*n* = 132, 78%) and the mean age was 40 (SD = 11.65 years). Regarding the occupational status, 22 were students (14.3%), four were first-time job-seekers (2.6%), 98 were employed (63.6%), and 30 were unemployed due to the disease and receive a disability benefit (19.5%). According to the current clinical criteria [1], 89 individuals received a diagnosis of cEDS (57.1%), 47 were diagnosed with hEDS (30.1%), three were diagnosed as vEDS (1.9%), and 17 were classified as sEDS (10.9%). Gender distribution was different among clinical subtypes (Exact Fisher’s test = 10.1, *p* = 0.013), and the post-hoc standardized residuals (st.res) revealed that hEDS is significantly less frequent in males (8.5% of hEDS were male, st.res = −2.0). Disability benefits were equally distributed among different clinical subtypes (Exact Fisher’s = 2.208, *p* = 0.508). Mean age at diagnosis (sEDS was excluded from the computation) was 35 years (SD = 12.39 years) with no difference between clinical subtypes (F = 1.157, df = 2, *p* = 0.317); age at symptoms appearance was different between groups (Exact Fisher’s = 27.173, *p* = 0.002) with symptoms appearing more frequently during childhood for hEDS (in 68.1% of the hDES subjects, symptoms appeared between 4 and 12 years of age, st.res = 1.96). As attended, the absence of luxation or subluxation was very rare in hEDS (st.res = −2.4).

No difference in pain was recorded among subgroups at the last assessment (mean VAS = 4.2/10, SD = 2.59, F = 2.131, df = 3, *p* = 0.099). The mean GHQ-12 Likert score was 19.46 (SD = 4.81). Using a cut-off ≥14, GHQ-12 was suggestive for a common mental disorder in 142 individuals (91%). The AQ score exceeded the cut-off (≥32) in 11 individuals (7.05%). Mean scores of WHOQOL-BREF domains were indicative of impaired QoL: Physical health mean 45.56 (SD = 22.41), psychological mean 55.05 (SD = 19.50), social relationship mean 53.37 (SD = 24.24), and environment mean 54.43 (SD = 17.27). QoL was impaired (scored <70) in the physical health domain for 127 individuals (81.4%), for 115 individuals (73.71%) in the psychological domain, for 115 individuals (73.71%) in the social relationship domain, and for 130 individuals (83.33%) in the environment domain. Considering the increasingly impaired QoL (defined as at least 1 SD below the sample mean), we identified 28 individuals (17.94%) in the physical health domain, 30 individuals (19.23%) in the psychological domain, 30 individuals (19.23%) in the social relationship domain, and 23 individuals (14.74%) in the environment domain. Table 1 presents the psychometric results. 

**Figure 1:**
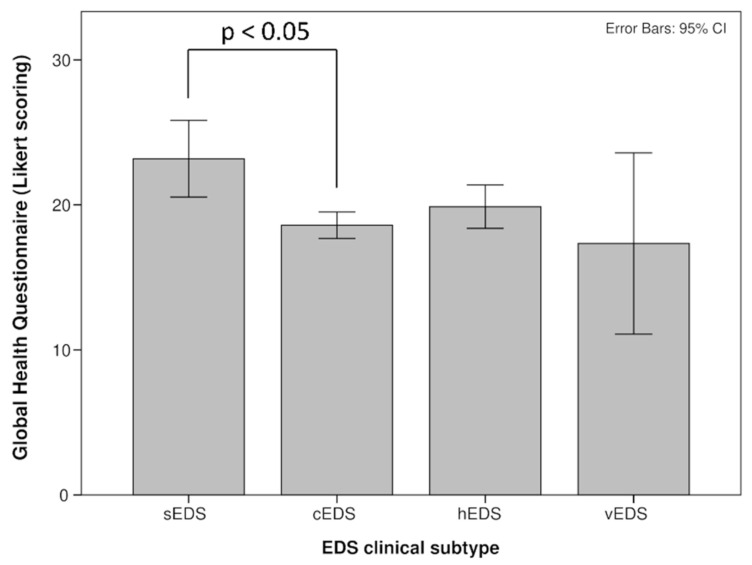
Global health questionnaire differences between EDS subtypes.

The EDS subgroups were scored differently at GHQ-12, with sEDS presenting the higher mean score and all of the individuals exceeding the common mental disorder cut-off (Figure 1).

**Figure 2:**
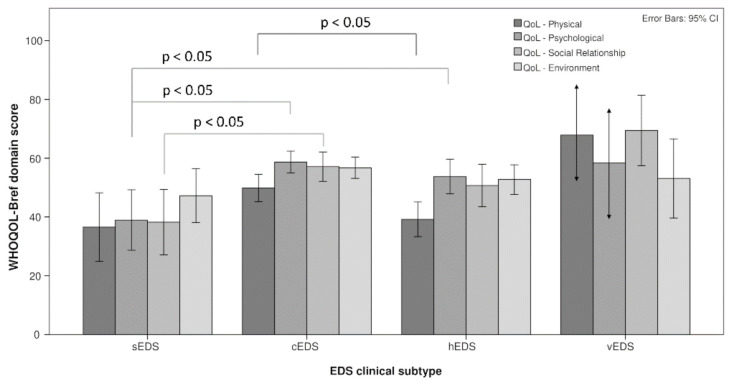
Bonferroni-corrected differences in WHOQOL-BREF domain scores among EDS subtypes.

Regarding the WHOQOL-BREF, a significant difference in the general self-rated QoL emerged. Specifically, subjects with sEDS scored lower than cEDS in the self-rated general QoL (mean difference −0.804, 95% CI from −1.46 to −0.15). Subgroups scored significantly different in the physical health domain, with hEDS subjects scoring lower than cEDS (mean difference −10.67, 95% CI from −0.22 to −21.12). Moreover, the psychological domain presented a significant between-group difference with sEDS scoring lower than both cEDS (mean difference −19.74, 95% CI from −32.98 to −6.50) and hEDS (mean difference −14.75, 95% CI from −28.91 to −0.60). Furthermore, scores in the social relationship domain were different among the subtypes. Specifically, sEDS scored lower than cEDS (mean difference −18.88, 95% CI from −35.60 to −2.17; see Figure 2). A severe impairment of QoL in the physical domain is less frequent in cEDS (st.res. −2.0), while in the psychological domain, sEDS presented a more frequent severe impairment of QoL (st.res. 2.6).

Age was negatively correlated with Environment QoL, with no significant correlation with the other QoL domains, pain, GHQ-12, and AQ. Pain was positively correlated with the GHQ-12 score and negatively correlated with each QoL domain. No correlation emerged between pain and AQ. GHQ-12 was negatively correlated with each QoL domain and positively correlated with AQ. AQ was negatively correlated with each QoL domain (see Table 2).

## 4. Discussion

This study represents the largest internet-based survey conducted in Italy to investigate the psychological distress, the QoL, and the presence of autistic traits in people diagnosed with EDS. Overall, people suffering from EDS appeared to be at risk for common mental disorders. Specifically, there was no risk difference between the diagnostic subtypes. However, the sEDS group (with a provisional diagnosis) showed the highest psychological distress. This is in line with the data regarding the psychological suffering derived from the uncertainty of the diagnosis [6]. Additionally, it seems particularly relevant in our sample, in which the age at diagnosis was high (35 years). Considering that most of the mental disorders emerged before the age of 24 [31] and that the higher delay in diagnosis is associated with a greater risk of attempted suicide [2], a careful psychiatric assessment should be recommended in people undergoing the diagnostic assessment for EDS, especially when a defined diagnosis is not reached. Considering the early symptom appearance in people with hEDS, as compared with the other subgroups, a diagnosis that remains purely clinical without the possibility for a genetic testing, it is not surprising that this EDS subtype has shown a significant high rate of psychological distress [7,32]. For this reason, psychological support should be offered especially for sEDS and hEDS.

Relevant ASD traits have been found in 7.1% of the sample. This is in line with a large population-based study [2] and is significantly larger than the most recent probabilistic estimate of ASD prevalence in Italy, which is 1.15% [33]. This factor should be further investigated with a multi-disciplinary assessment for ASD, the gold standard for autistic traits in adulthood [34]. If confirmed, the high prevalence of ASD in EDS should suggest a shared etiological hypothesis [35,36]. Moreover, the co-occurrence of EDS and ASD have a clinical implication. For instance, it has been associated with fatigue and sleep problems in children [15]. This is in line with our findings, which showed a significant positive association between autistic traits and psychological distress and a significant negative correlation between AQ and each QoL domain. Since the presence of comorbid ASD could reduce the validity of screening tools for psychological distress in the general population [37], our results appeared even more striking, advocating special attention for this possible comorbidity.

Overall, the global functioning of people with EDS is significantly hampered, as shown by the high prevalence of unemployment status due to the disease and by the high proportion of people receiving a disability benefit (19.5%), with no significant difference according to the clinical subgroups.

Pain recorded at the last visit before the survey was relatively low and no difference emerged between the subgroups. Greater pain was significantly associated with a more severe psychological distress (higher GHQ-12 scores), and reduced quality of life. No correlation emerged with autistic traits. Given the observational nature of this study, we could only provide a hypothesis on the direction of the association. It is possible that pain aggravates the psychological burden in EDS [14], but at the same time patients could develop a more resilient psychological status, through psychological support, that ameliorates pain management in EDS [7].

Quality of life was significantly impaired in our sample, with less than 30% of the sample considering their QoL as good or very good. Almost 20% of the subjects revealed a severely impaired quality of life in mental health and social functioning. This finding is consistent with Morlino et al. [38] who found a significant reduction in QoL and a significant association with functional difficulties. Additionally, they observed that the number of painful joints was a predictor of functional impairment. In Italy, impaired QoL was already reported in the small sample of 21 subjects with EDS [19]. However, only physical QoL was impacted with no effect on mental health. Several subsequent studies, however, have underlined the significant reduction in mental health-related QoL in this patient group [17,32,39]. In fact, a recent meta-analysis highlighted the role of psychological symptoms in aggravating the disability in hypermobility syndromes [8].

Interestingly, further differences emerged among the different diagnostic subgroups. Whereas hEDS reported lower health-related QoL, the sEDS showed the lowest mental and social-relationship QoL. These findings support the hypothesis that not being able to have a definite diagnosis worsens the psychological burden of people with EDS, probably affecting also some aspect of their social life [6].

### Limitations

A few limitations in our observational study need to be acknowledged. The recruitment through a tertiary center could have limited the observation to patients with a more severe clinical condition than the average EDS patients. Additionally, only about 30% of the patients answered the internet-based survey. This response rate is in line with another web-based survey [40]. However, we could not exclude the possibility that the people that agreed to participate were more prone to report psychological discomfort. Finally, given the observational nature of this study, no causal inference could be made on the possible determinants of the severe impairment in QoL, psychological wellbeing, and the high prevalence of autistic traits.

## 5. Conclusions

Our study showed a high level of psychological distress, significant worsening in all of the quality-of-life domains, and a higher prevalence of autistic traits in EDS. Pain appeared to be associated with psychological distress and a worse quality of life. These findings support our claim for a multi-disciplinary approach and integrated care in EDS, with a specific attention to psychological issues in this patient group.

## Figures and Tables

**Table 1:** Mean GHQ-12, AQ, and WHOLQOL-BREF scores with individuals exceeding the cut-off.

Assessment Tool	Total	EDS Subtype				F (df, *p*-Value)	Fisher’s (*p*-Value)
		sEDS	cEDS	hEDS	vEDS		
		17 (10.9%)	89 (57.1%)	47 (30.1%)	3 (1.9%)		
**GHQ-12 Likert (SD)**	19.46 (4.81)	23.18 (5.14) *	18.60 (4.31)	19.87 (5.08)	17.33 (2.52)	**5.021** **(3, *p* = 0.002)**	
*n* (%) with GHQ-2 score ≥ 14	142 (91.0%)	17 (100%)	78 (87.6%)	44 (93.6%)	3 (100%)		n.s.
**AQ (SD)**	19.99 (6.80)	22.65 (6.86)	19.57 (6.29)	20.19 (7.62)	14.33 (3.51)	1.705 (3, *p* = 0.168)	
*n* (%) with AQ score ≥ 32	11 (7.1%)	2 (11.8%)	5 (5.6%)	4 (8.5%)	0		n.s.
**WHOQOL-General QoL(self-rated) (SD)**	3.00 (0.95)	2.35 (0.79) *	3.16 (0.95)	2.98 (0.92)	2.33 (0.58)	**4.182** **(3, *p* = 0.007)**	
**WHOQOL-Health satisfaction(self-rated) (SD)**	2.46 (1.00)	2.12 (0.78)	2.57 (1.00)	2.38 (1.05)	2.00 (1.00)	1.359 (3, *p* = 0.258)	
**Physical Health (SD)**	45.56 (22.41)	36.55 (22.75)	49.88 (22.11)	39.21 (20.06) *	67.86 (28.57)	**4.557** **(3, *p* = 0.004)**	
*n* (%) with domain score < 70	127 (81.4%)	15 (88.2%)	68 (76.4%)	42 (89.4%)	2 (66.7%)		n.s.
*n* (%) with severe impairment	28 (17.9%)	6 (35.3%)	*8 (9.0%)* *	14 (29.8%)	0		**12.943** **(*p* = 0.003)**
**Psychological (SD)**	55.05 (19.50)	38.97 (19.98) *	58.71 (17.70)	53.72 (20.01)	58.33 (20.83)	**5.427** **(3, *p* = 0.001)**	
*n* (%) with domain score < 70	115 (73.7%)	14 (82.4%)	62 (69.7%)	37 (78.7%)	2 (66.7%)		n.s.
*n* (%) with severe impairment	30 (19.2%)	8 (47.1%) *	12 (13.5%)	10 (21.3%)	0		**9.427** **(*p* = 0.018)**
**Social Relationship (SD)**	53.37 (24.24)	38.24 (21.66) *	57.12 (23.69)	50.71 (24.62)	69.44 (4.81)	**3.734** **(3, *p* = 0.013)**	
*n* (%) with domain score < 70	115 (73.7%)	16 (94.1%)	62 (69.7%)	35 (74.5%)	2 (66.7%)		n.s.
*n* (%) with severe impairment	30 (19.2%)	7 (41.2%)	13 (14.6%)	10 (21.3%)	0		n.s.
**Environment (SD)**	54.43 (17.27)	47.24 (17.85)	56.74 (17.18)	52.73 (17.20)	53.13 (5.41)	1.694 (3, *p* = 0.171)	
*n* (%) with domain score < 70	130 (83.3%)	15 (88.2%)	72 (80.9%)	40 (85.1%)	3 (100%)		n.s.
*n* (%) with severe impairment	23 (14.7%)	5 (29.4%)	7 (7.9%)	11 (23.4%)	0		**9.226** **(*p* = 0.018)**

Legend: AQ: Autism quotient; EDS: Ehler Danlos Syndrome (c: Classical; h: Hypermobile; s: Suspected; v: Vascular); GHQ: Global Health Questionnaire; QoL: Quality of Life; SD: Standard deviation; WHOQOL: World health Organization Quality of Life assessment tool. Bold values refer to significant F statistics or Fisher’s exact test. Italic with * refers to significant post-hoc comparisons corrected with Bonferroni’s method. Cronbach’s α >0.7 for each assessment tool.

**Table 2:** Pearson’s correlation matrix.

Clinical Variable		Age	Pain (VAS)	GHQ-12 Likert	QoL-Physical Health	QoL-Psychological	QoL-Social Relationship	QoL-Environment
**Age**	r	-						
**Pain (VAS)**	r	0.154	-					
**GHQ-12 Likert**	r	0.124	0.195 *	-				
**QoL-Physical Health**	r	−0.144	−0.501 **	−0.523 **	-			
**QoL-Psychological**	r	−0.059	−0.183 *	−0.625 **	0.65 6 **	-		
**QoL-Social relationship**	r	−0.138	−0.283 **	−0.417 **	0.483 **	0.541 **	-	
**QoL-Environment**	r	−0.205 *	−0.365 **	−0.503 **	0.685 **	0.587 **	0.493 **	-
**AQ**	r	−0.084	0.050	0.326 **	−0.320 **	−0.382 **	−0.350 **	−0.334 **

Legend: AQ: Autism quotient; GHQ: Global Health Questionnaire; QoL: Quality of Life. * = Two-tailed *p* < 0.05; ** = two-tailed *p* < 0.01.

## Data Availability

Data will be available upon reasonable request from the first author.

## Supplementary Materials

The following are available online at www.mdpi.com/article/10.3390/healthcare9111472/s1. Table S1: CHERRIES guidelines assessment.
